# Supplementary material for: Acute Effects of Ocrelizumab Infusion in Multiple Sclerosis Patients
Source: Int J Mol Sci. 2022 Nov 9;23(22):13759. doi: 10.3390/ijms232213759 (PMC9696175; doi:10.3390/ijms232213759)
Supplement: Supplementary file 1 [file ijms-23-13759-s001.zip › ijms-1937007-supplementary.pdf]

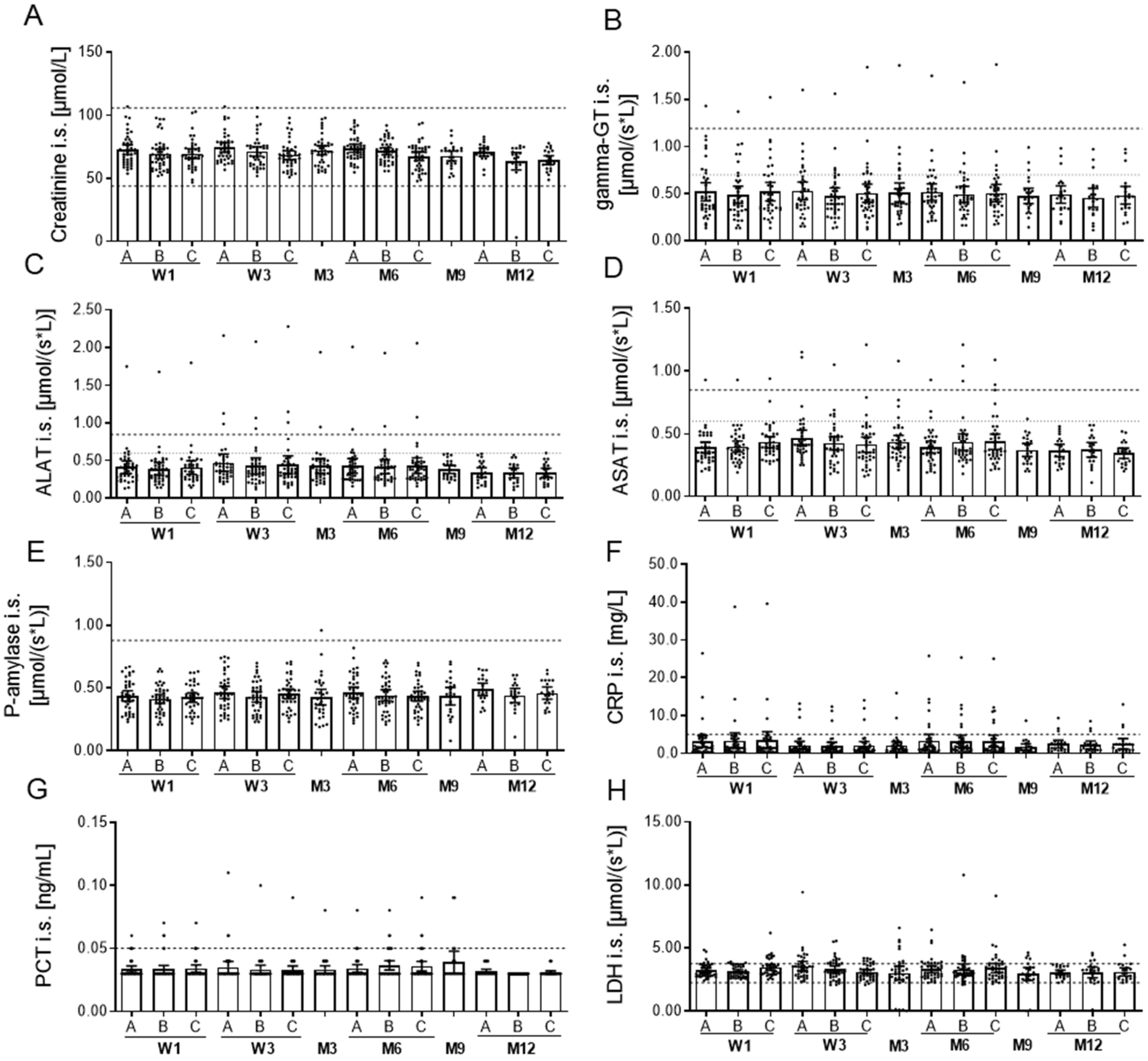

Figure S1. Clinical chemistry during OCR infusions. Blood samples were taken before infusions started [A], after MP [B], and after OCR [C] on each at the first two OCR infusions at week 1 (W1) and week 3 (W3), third infusion at month 6 (M6) and fourth infusion at month 12 (M12). Additional blood controls were done at month 3 (M3) and month 9 (M9). Dotted lines mark reference range of depicted parameters. Bar/scatter plots with mean with 95%CI are depicted. (A, E-G, H) Dotted line indicates the reference range. (B-D) Bold dotted line indicates the reference range for males, fine dotted line for females. Asterisks indicate a statistically significant difference between selected time points and global effects (* $p < 0.05$, ** $p < 0.01$, *** $p < 0.001$).

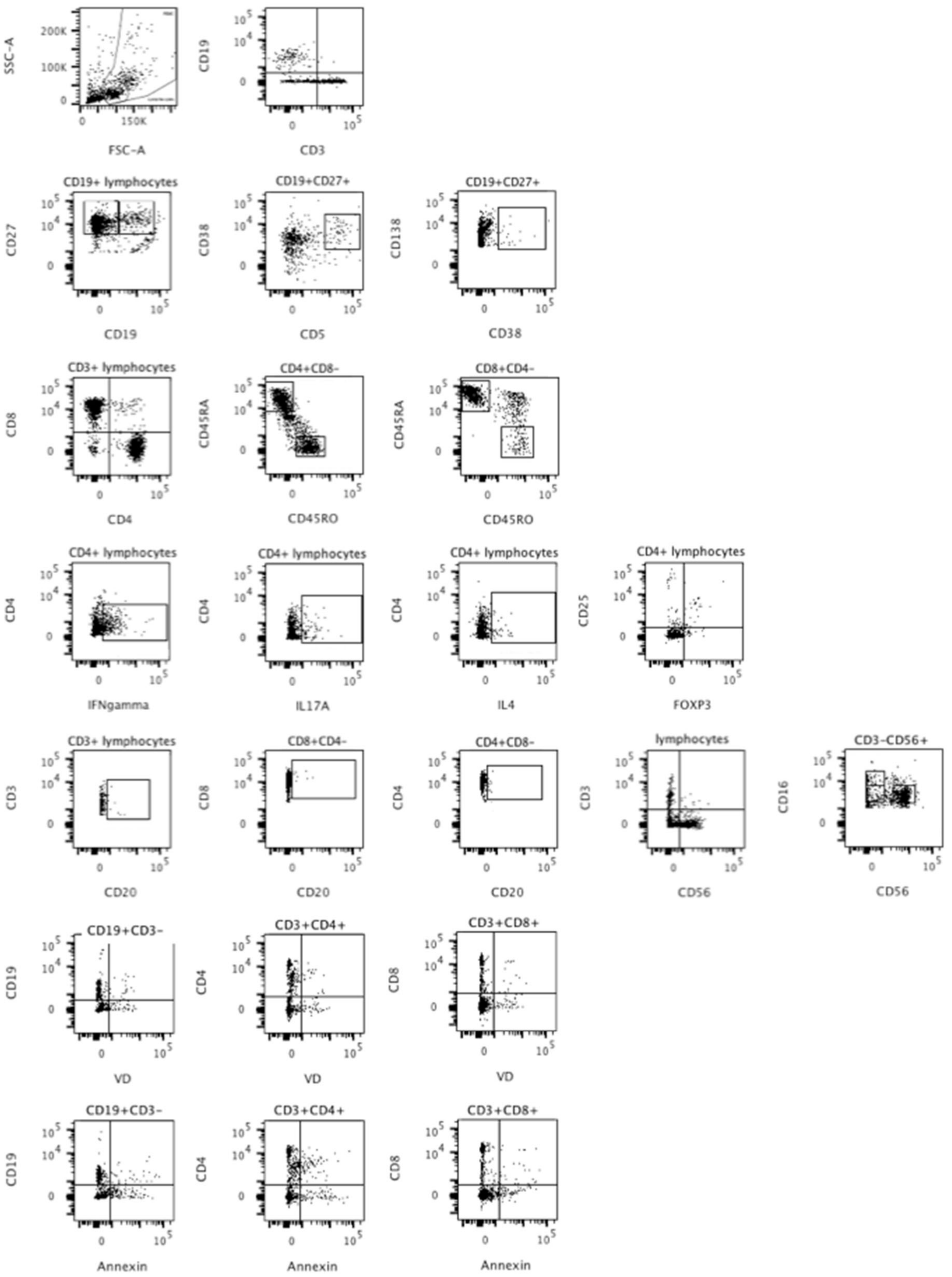

Figure S2. Gating strategy of analyzed peripheral immune cell subsets evaluated by FACS analysis. The following subtypes were included: CD19+CD27- naïve B cells, CD19+CD27+ memory B cells, CD19+CD27+CD38+CD138+ plasmablasts, CD19+CD27+CD38+CD5+ B regulatory cells, proportion of CD4+ and CD8 T cells including naïve (CD45RA+) and memory (CD45RO+) subsets, IFN-gamma+ CD4+ Th1 cells, IL17A+ CD4+ cells, IL4+ CD4+ Th2 cells, CD4+CD25+FOXP3+ Treg cells, CD3-CD56+ NK cells, CD3+CD56+ NKT cells, NK cell subsets (CD56^{low}CD16^{-}; CD56^{low}CD16^{bright}; CD56^{bright}CD16^{-}), apoptotic (annexin, A) and death (viability dye, VD) B and T cells. Monocyte subsets (classical, intermediate, non-classical monocytes) and slanDCs, myeloid and plasmacytoid DCs did not present any changes and though are not presented here but are available by request.
